# Functional Proteomic Profiling of Secreted Serine Proteases in Health and Inflammatory Bowel Disease

**DOI:** 10.1038/s41598-018-26282-y

**Published:** 2018-05-18

**Authors:** Alexandre Denadai-Souza, Chrystelle Bonnart, Núria Solà Tapias, Marlène Marcellin, Brendan Gilmore, Laurent Alric, Delphine Bonnet, Odile Burlet-Schiltz, Morley D. Hollenberg, Nathalie Vergnolle, Céline Deraison

**Affiliations:** 1 IRSD, U1220, Université de Toulouse, INSERM, INRA, ENVT, UPS, Toulouse, France; 2 Institut de Pharmacologie et de Biologie Structurale, Université de Toulouse, CNRS, UPS, Toulouse, France; 3 School of Pharmacy, Queen’s University, Belfast, Ireland; 4 Pôle Digestif, CHU Purpan, Toulouse, France; 5 Department of Physiology and Pharmacology, Faculty of Medicine, University of Calgary, Calgary, Alberta Canada

## Abstract

While proteases are essential in gastrointestinal physiology, accumulating evidence indicates that dysregulated proteolysis plays a pivotal role in the pathophysiology of inflammatory bowel disease (IBD). Nonetheless, the identity of overactive proteases released by human colonic mucosa remains largely unknown. Studies of protease abundance have primarily investigated expression profiles, not taking into account their enzymatic activity. Herein we have used serine protease-targeted activity-based probes (ABPs) coupled with mass spectral analysis to identify active forms of proteases secreted by the colonic mucosa of healthy controls and IBD patients. Profiling of (Pro-Lys)-ABP bound proteases revealed that most of hyperactive proteases from IBD secretome are clustered at 28-kDa. We identified seven active proteases: the serine proteases cathepsin G, plasma kallikrein, plasmin, tryptase, chymotrypsin-like elastase 3 A, and thrombin and the aminopeptidase B. Only cathepsin G and thrombin were overactive in supernatants from IBD patient tissues compared to healthy controls. Gene expression analysis highlighted the transcription of genes encoding these proteases into intestinal mucosae. The functional ABP-targeted proteomic approach that we have used to identify active proteases in human colonic samples bears directly on the understanding of the role these enzymes may play in the pathophysiology of IBD.

## Introduction

The degradome represents almost 2% of protein coding genes in the human genome, with at least 588 genes coding for proteases. Among them, one of the largest classes is represented by 184 genes encoding serine proteases, which are characterized by the presence of a nucleophilic serine in their reactive site^1^. Since the hydrolysis of peptide bonds is an irreversible process, the expression and activity of proteases are tightly regulated. For instance, these enzymes often exist as inactive zymogens (pro-forms), which must be activated by proteolytic cleavage. A large array of endogenous protease inhibitors also exists that can control cell and tissue proteolysis.

Proteases are essential mediators in gastrointestinal physiology, being produced and released by the pancreas, in order to be activated in the intestinal lumen for digestive purposes. Proteolytic activity is also detected within mucosal tissues in healthy conditions and is thought to play a role in mucus consistency and mucosal antigen processing^2^. Otherwise, in intestinal pathophysiological contexts such as inflammatory bowel disease (IBD), proteolytic homeostasis can be disrupted in tissues^2^. Increased serine protease activity has been demonstrated in colonic tissues from Crohn’s disease (CD) or Ulcerative Colitis (UC) patients^3–5^. Some of these studies also demonstrated that the reestablishment of the proteolytic homeostasis by the local delivery of recombinant protease inhibitors reduces the severity of experimentally-induced colitis^3,6^, thus highlighting the importance of these enzymes both as central mediators of IBD pathophysiology, and as potential therapeutic targets.

The identity of overactive serine proteases in intestinal tissues remains elusive. *In situ* zymography assays demonstrated that the increased IBD-associated elastolytic activity was mostly present within the epithelium^3^. This is an interesting finding, given that most studies aimed at identifying upregulated proteases in inflammatory diseases have focused on enzymes highly expressed by infiltrating immune cells. Thus, gene and protein expressions of several proteases released primarily by leukocytes (including neutrophil elastase, proteinase-3, cathepsin G, tryptase, chymase or granzymes) have been found to be upregulated in IBD^2^. Additionally, genetic studies have supported an association of protease genes with IBD risk^7,8^. Nevertheless, the major limitations of such studies based on expression analysis are due to the fact that mRNA or protein levels for proteases do not necessarily reflect their activity status. Indeed, variations of zymogen activation or local availability of endogenous inhibitors can drastically modify biological activity.

Therefore, the identity and implication of proteases in health and diseases, including IBD, have to come from studies investigating the *in situ* net activity of these enzymes^9^. The development of functional proteomic assays based on Activity-Based Probes (ABPs) now allows such approaches, monitoring the availability of enzyme active sites in biological samples^10–13^.

The ABP structure possesses a reactive group that mimics enzymatic substrate and covalently binds to active proteases. Additionally, the ABP reactive group is associated to a biotin motif *via* a spacer, in such a way that bound active enzymes thus become biotinylated and can be visualized and/or immobilized by avidin-based affinity chromatography. Further mass spectral analysis could then determine the enzyme sequence. Obviously, detection of active proteases is dependent on their affinity towards the ABP that is used. We have previously used this approach successfully to identify active serine proteases upregulated in the setting of a murine model of infectious colitis^14^ and to determine the sequences of serine proteases present in complex allergenic cockroach extracts^15^. Here, we performed a study to profile and identify active serine proteases secreted by the colonic mucosa of control and IBD patients by using ABPs.

## Results

### Validation of the sensitivity for detecting Trypsin-like activity using a Biotin-PK-DPP activity-based serine protease probe: signal intensity correlates with trypsin activity level

**Figure 1:**
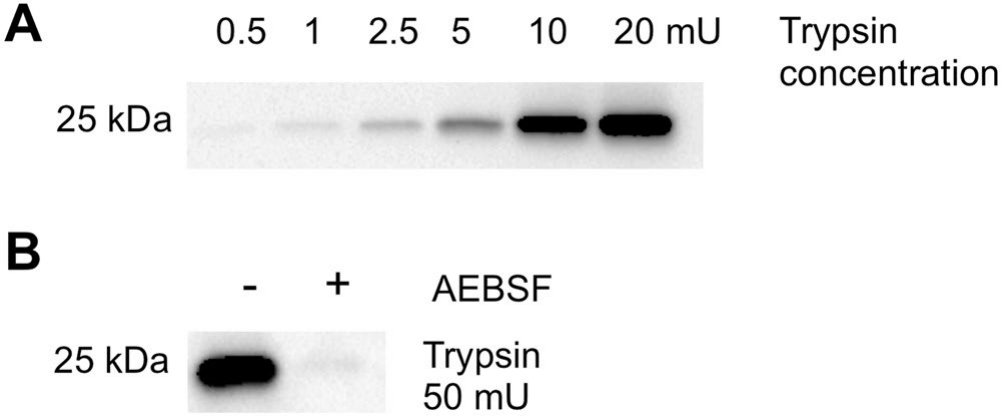
Validation of biotin-PK-DPP sensitivity for detection of trypsin-like enzymes (**A)**. 1 µM PK-ABP was incubated with an increasing concentration of trypsin and the biotinylated trypsin product was visualized by electrophoresis followed by detection using streptavidin-linked horseradish peroxidase and ECL. (**B**) Trypsin was treated first with the broad-spectrum serine protease inhibitor AEBSF (4 mM) (the + condition) prior to its reaction with ABP and ECL detection.

The ABP biotin-PK-DPP synthesized for the present study^16^ was of sufficient reactivity to detect a level of 2.5 mU of trypsin from bovine pancreatic trypsin. The ABP signal intensity was proportional to increasing concentrations of trypsin, and was eliminated by the serine protease irreversible inhibitor AEBSF (Fig. 1A, B and Supplementary Figure 1).

### Secreted serine protease activity is upregulated in IBD colonic mucosa

**Figure 2:**
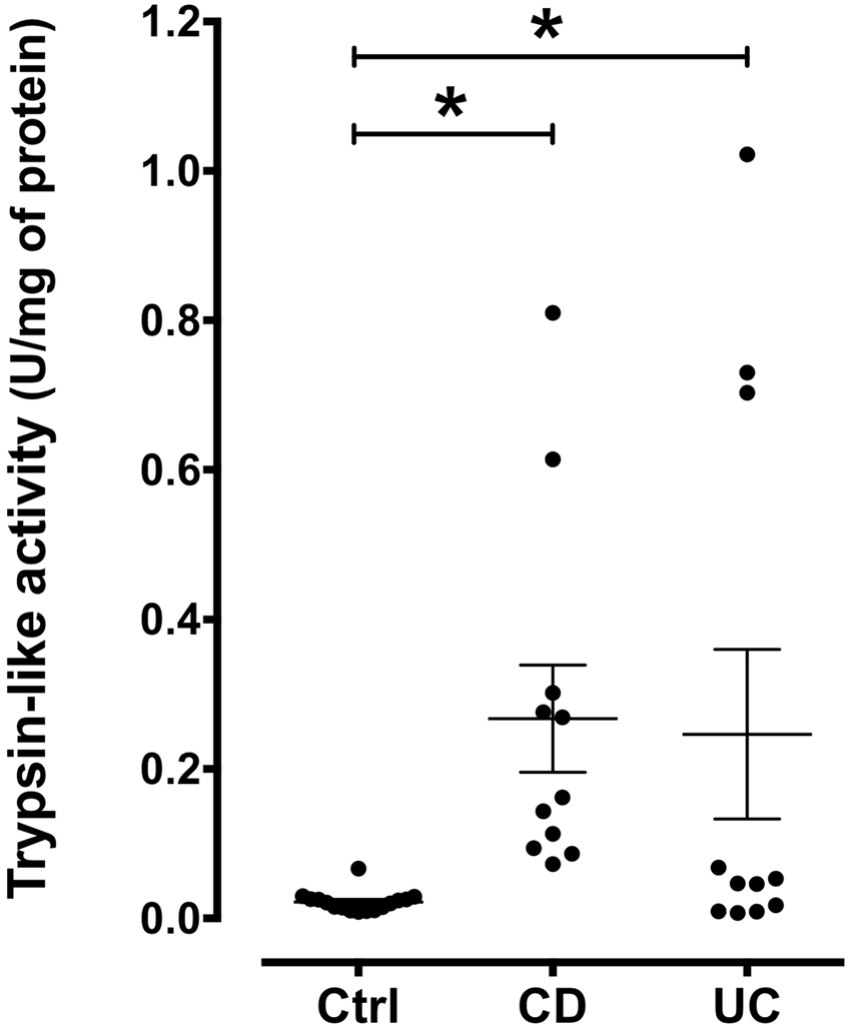
Measurement of trypsin-like activity released by human colonic mucosa. Trypsin-like activity detected in supernatants from colonic tissue samples of control or IBD patients (n = 11–16). Data were analysed by ANOVA followed by the multi comparison test of Holm-Sidak. *P < 0.05 *vs*. control.

**Figure 3:**
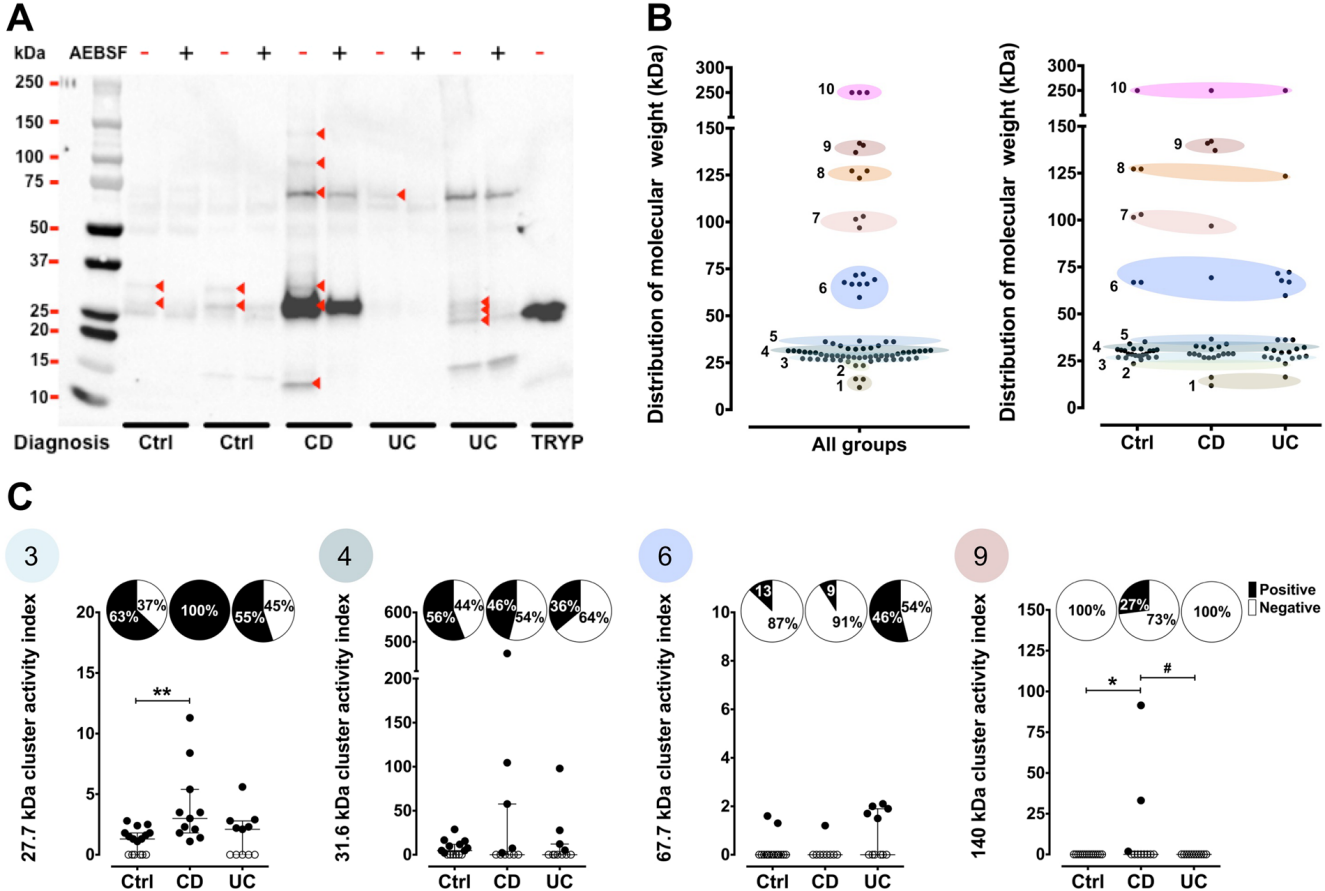
Proteomic profiling of serine proteases released by the human colonic mucosa. (**A**) Representative ABP proteomic profile, showing the differential repertoire of ABP-labelled serine proteases secreted from control or IBD colonic tissue samples along with the positive trypsin control (20 mU of trypsin). The red arrowheads point to bands corresponding to active proteases, as verified by the inhibitory effects of pre-treatment of the samples with AEBSF (4 mM). (**B**) Clustering of ABP-labelled serine proteases according to size (kDa). (**C**) Graphic representation of protease size clusters along with their activity index determined by the impact of enzyme inhibition (−/+AEBSF). The percentage of AEBSF-inhibited bands per patient is represented by the pie graphs. The empty circles represent patients wherein bands within the cluster were not detected (negative), a 0 value was given to these samples as per their activity index. Activity index data were analysed by Kruskal-Wallis followed by the multi comparison test of Dunn. *P < 0.05, **P < 0.01, *vs*. control; ^#^P < 0.05 *vs*. CD.

Colonic tissue supernatants from control patients exhibited a baseline proteolytic activity, which was increased in samples from CD and UC patients (Fig. 2). To characterize the serine proteases underlying this increase of proteolytic activity, we initially performed ABP proteomic profiling assays with these samples. Since ABPs react only with active enzymes, the bands corresponding to proteases were discriminated from non-specific labelling by pre-incubating the samples in parallel with the serine protease inhibitor, AEBSF. Therefore, the signal intensity of protease bands from AEBSF-treated samples was reduced or absent in comparison with the sample not treated with this irreversible serine protease inhibitor. As a whole, bands representing putative serine proteases ranged from 12 to 250 kDa (Fig. 3A). A distribution analysis of putative proteases according to their molecular weight regrouped them in 10 main clusters, with mean molecular weights of 15, 24, 28, 32, 36, 68, 100, 126, 140 and 250 kDa. The majority of serine proteases were grouped into the 28, 32 and 36 kDa clusters (Fig. 3B). Once these clusters were analysed in individual groups of patients, some differences became evident. Likewise, the cluster 1 (15 kDa) was only detected in IBD samples. The cluster 6 (68 kDa) was more prominent in UC samples, while cluster 9 (140 kDa) was only detected in CD samples.

Next, we focused on the number of AEBSF-sensitive bands, which were diminished in the presence of the protease inhibitor, as opposed to the labelled bands which were not affected by AEBSF incubation. In all samples there were biotin-labelled constituents in the 50 and 65 kD range which appeared to yield a comparable streptavadin-biotin reactivity, for which the signal was not diminished by AEBSF treatment (Fig. 3A). Relative to those AEBSF-resistant signals present in all samples, quite distinct AEBSF-sensitive ABP labelling profiles were observed for samples obtained from the CD and UC individuals either compared between diseases or compared to controls (Fig. 3A). In particular, the CD-derived samples contained a unique ABP-labelled constituent in the 15 kDa range comparable to a labelled component found in the trypsin preparation, that might represent a cleaved, catalytically active fragment of trypsin (Supplementary Figure 1). Other higher molecular mass AEBSF-sensitive ABP-labelled bands also distinguished the CD samples from the UC and control samples. This distinction was quantified further by analysing the AEBSF-sensitivity of labelled bands for constituents clustered in the mass regions of 28 (cluster 3), 32 (cluster 4), 68 (cluster 6) and 140 (cluster 9) kDa. According to this analysis, differences in the percentage of AEBSF-sensitive bands present in control *versus* CD samples were observed for clusters 3 (28 kDa: 63% reduction in Controls *vs*. 100% inhibition by AEBSF, in CD) and 9 (140 kDa: 8% reduction in Controls *vs*. 25*%*, in CD). Changes in the percentage of AEBSF-sensitive bands between controls and UC were observed for cluster 6 (68 kDa; 13% reduction caused by AEBSF in Controls *vs*. 42% reduction in UC) (Fig. 3C).

We then defined the activity index of each cluster bands by considering the intensity in the absence *versus* in the presence of AEBSF (Fig. 3C). In control samples, the most active cluster was at 32 kDa (Fig. 3A,C, cluster 4). The comparison of the activity index between control and IBD samples revealed an increased proteolytic activity index associated with some clusters. For instance, the activity index for clusters 3 and 9 (28 and 140 kDa, respectively) increased in CD samples. That said, although for bands in the 32 and 68 kDa (clusters 4 and 6) a number of AEBSF-sensitive labelled bands appeared to differ between the CD and UC-derived samples, the difference in the activity index did not quite reach statistical significance (Fig. 3C).

### ABP-reactive enzymes identified by LC-MS/MS analysis

Unbiased mass spectrometric analysis identified 6 proteases from S1 family in samples from colonic biopsy supernatants. These proteases were considered active according to the ability of AEBSF to block labelling, with an activity index >2 and P < 0.05 (Table 1). Here, the activity index was defined by the ratio −/+AEBSF of the quantity of positive peptides identified by LC-MS-MS analysis. This group of identified active proteases includes thrombin, cathepsin G, kallikrein-1 (also named plasma kallikrein), plasmin, chymotrypsin-like elastase family member 3A and tryptase. Additionally, aminopeptidase B (also called arginyl aminopeptidase), a lysine-cleaving protease from the M01 family was also identified as active. Overall, thrombin was the most active protease identified, and its activity was particularly prominent in CD. Similarly, aminopeptidase B was highly active specifically in association with UC (Table 1).

**Table 1:** Active ABP-labelled serine proteases secreted from the colonic mucosa of control and IBD patients.

Protease family	Gene symbol	Protein name	Predicted MW	Activity index		
				Ctrl	CD	UC
S01	F2	Thrombin−α/−β/−γ	32/28/15	<2	97.57	4.9
	CTSG	Cathepsin G	29	<2	n.d.	5.8
	KLKB1	Plasma kalikrein	71	4.2	<2	5.1
	PLG	Plasmin	91	2.5	<2	2.3
	TPSAB1/B2	Tryptase−α/β1/−β2	31/30	2.3	2.03	<2
	CELA3A	Chymotrypsin-like elastase family member 3A	30	2.8	<2	<2
M01	RNPEP	Aminopeptidase B	73	<2	<2	19

The list shows the active ABP-labelled proteases identified by LC-MS/MS analysis of pooled supernatant samples from control and IBD patients, showing the respective protease family, gene symbol, protein name, predicted molecular weight and the activity index reflecting the sensitivity of ABP labelling to protease inhibition (−/+AEBSF ratio).

### Active secreted proteases identified by ABP labelling are expressed by the intestinal mucosae

**Figure 4:**
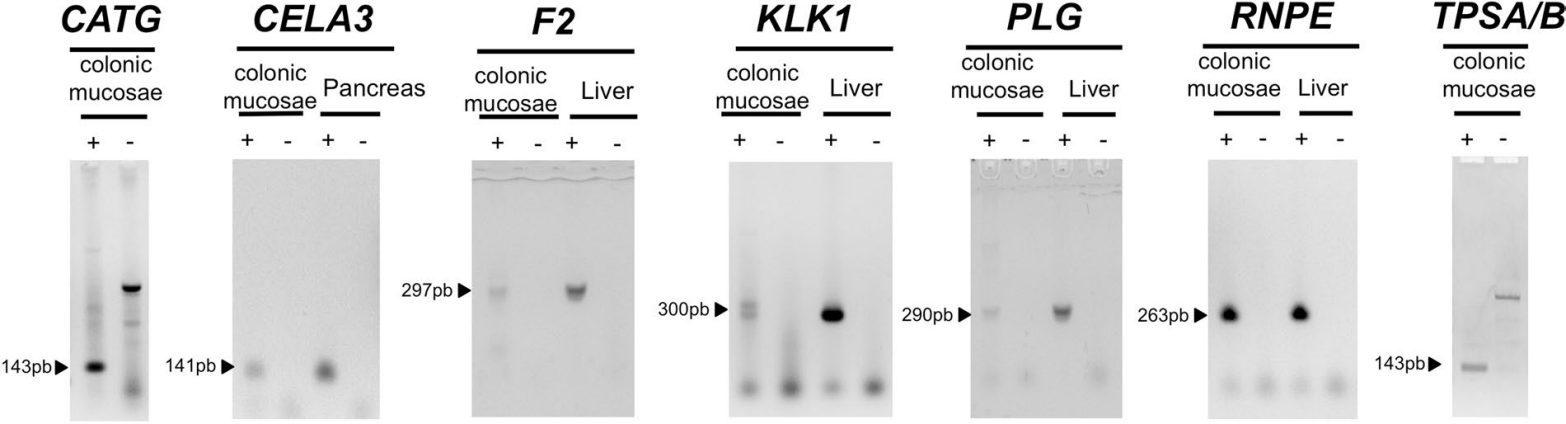
Colonic RNA expression for proteases identified as active. Analytical agarose-gel electrophoresis of RT-PCR products amplified from cDNAs prepared from human colonic mucosa tissue samples are shown with arrows denoting the predicted size (base pairs: bp) of the PCR product. Negative controls (noted -) consisted of RT reactions performed in the absence of enzyme. Positive expression was confirmed using cDNAs prepared from human tissue sources known to express the target proteases.

Gene expression experiments were carried out to investigate whether or not the proteases identified as active were expressed in the human colonic mucosa. RT-PCR products were detected for the 7 proteases, wherein amplicons with expected base pair numbers were amplified from colonic mucosa (Fig. 4).

## Discussion

Mass spectrometry proteomic approaches have been applied to IBD tissues, identifying global changes in proteome for these pathologies^17–20^. Using such approaches, only few proteases were identified. Their relative abundance seems to be secondary to immune cell infiltration as they are major components of innate immune cells. As a matter of course, the major drawback of classical proteomic approaches remains the lack of information about protease activity. As a consequence, the implication of these enzymes in the pathophysiology of human diseases has been only marginally characterized to date. Herein, we used a biotinylated ABP capable of interacting with lysine-cleaving proteases (biotin-PK-DPP), a catalytic feature of most serine proteases and some proteases from other classes^21^. Furthermore, experiments performed with increasing amounts of active trypsin clearly demonstrated that the signal intensity generated by this ABP augmented accordingly, thus highlighting that this probe can unveil varying activity levels of lysine-cleaving proteases. The ABP proteomic gel profiles revealed the presence of bands with a broad molecular weight distribution, either sensitive or not to AEBSF inhibition. Because streptavidin which is used to reveal biotinylated bands, can bind non-specifically to proteins^22,23^, and because we cannot fully exclude that ABP might bind non-specifically to some proteins in a complex mixture, the use of inhibitory AEBSF pre-treatment in counterpart samples was instrumental at discriminating active protease bands.

In previous work using activity-based probes to identify active serine proteases associated with intestinal inflammation in an infectious model of rodent colitis, we established a role for host serine proteases and their signalling target, protease-activated receptor-2 (PAR2), in driving the inflammatory response^14^. The results we report here establish proof of principle, that a comparable approach can be used to evaluate patient-derived tissue samples. Our work considerably extends our previous observations which showed that explants from individuals with IBD secrete increased lysine-targeted protease activity. Our main finding is that compared with non-diseased tissues, serine protease-targeted activity-based probes reveal a distinct set of active serine proteases secreted by colonic tissues derived from individuals with either Crohn’s disease or ulcerative colitis. These data amplify in molecular terms, the initial finding that the secretome of tissues derived from IBD patients contained increased trypsin-like activity, relative to controls. Our data also complement observations by others reporting the increased presence of cathepsin-G in faeces of patients with ulcerative colitis^5^.

Several studies have documented high levels of tryptase in IBD mucosae^24–26^. However, increased level of tryptase in IBD mucosae was reported based on immunoassays^24,26^. Active tryptase was not detected in the secretome from UC biopsies in this study. Our results suggest that concomitantly with exocytosis of tryptase, endogenous inhibitors could also be present in the granules or in the vicinity of activated mast cells, leading to a quick neutralization of enzymatic activity. While from protein or mRNA expression studies, tryptase could appear as a potential molecular target for IBD, our results suggest on the contrary that active tryptase is not present in patient’s tissues.

The ABP-tagged enzymes that were distinct in the secretome from the Crohn’s disease and ulcerative colitis-derived tissues, compared with disease-free tissues, fell into four clusters. One cluster represented by a protease in the 10 kDa range was found only in tissues from CD individuals (Fig. 3A) and three others, two of which (clusters 3 and 9, in the range of 28 and 140 kDa) were associated with Crohn’s disease and a fourth (cluster 6, in the range of 68 kDa), which was associated with samples from ulcerative colitis individuals. In addition, within these clusters, the scatter-plots, with groups of points well above baseline, suggest that a subset of individuals may be present within each cluster. It will be of importance to follow the clinical outcomes of the individuals with high activation profiles within the clusters, compared with the others in each patient group.

The expression of all serine proteases identified by ABP labelling, namely thrombin, cathepsin G, plasma kallikrein, plasmin, tryptase and chymotrypsin-like elastase family member 3 A, were also found as mRNA transcripts in extracts of colon biopsy tissue (Table 1 and Fig. 4). Thus, all of the ABP-labelled enzymes can be both produced and secreted *in situ* by mucosal tissue. Most of these identified serine proteases are well-established activators of Protease-Activated Receptors (PARs), which have been implicated in IBD pathophysiology^16,17^. Further, the enzymes like cathepsin G, in addition to regulating PAR activity, can play an inflammatory role *via* either the processing/activation of cytokines, chemokines and growth factors (e.g. the convertase action of cathepsin-G for generating alarmin-IL-33) or by the cleavage/inactivation of such mediators^27^. It will be important to validate whether or not the active proteases we have identified in the tissue secretome would also be found as active enzymes in fecal samples, so as to provide for a ‘biomarker’ to follow disease progression.

A high level of active thrombin was particularly detected in the secretome from CD mucosal biopsies and to a lesser extent from the ulcerative colitis-derived tissues. Biopsies from IBD patients were collected in macroscopic inflamed areas, where ulceration or erythema is observed, which can be associated to blood vessel leakage. At the site of colitis, circulating pro-thrombin could be activated by tissue factor Xa expressed on cells like monocytes, dendritic cells, platelets, endothelial cells and vascular smooth muscle cells. Of particular note, thrombin was also identified at colonic tissue mRNA transcript level. This extrahepatic source of thrombin could therefore also participate to modulation of innate immunity, notably via PARs 1 and 4 receptor cleavage. Activation of these receptors could result in intestinal epithelial cell apoptosis and barrier disruption^18^ and might cause either an inflammatory or anti-inflammatory response in the tissues^19^. The exact role that thrombin might play in Crohn’s disease and ulcerative colitis, diseases that have been associated with increased thrombosis^20,21^, remains to be clarified.

Surprisingly, one protease from a class other than serine protease was identified as active in samples from UC patients: the metallopeptidase aminopeptidase B. This enzyme is known to display endopeptidase activity after an Arg or a Lys residue^28^ and this could explain the fact that it binds to the ABP we have used here. To the best of our knowledge, the role of aminopeptidase B in IBD pathophysiology has not been evaluated yet.

In summary, the functional proteomic approach employed herein allowed for the identification of a consistent proteomic profile of active serine proteases secreted by the colonic mucosa of healthy and IBD patients. Additionally, the use of ABP labelling in conjunction with mass spectral proteomic analysis resulted in the identification of unique active proteases selectively secreted from the colitis-derived samples, compared with samples from disease-free individuals. This approach identified not only proteases previously established as putative candidates in IBD pathophysiology, but also enzymes not yet appreciated in this context. In this way, the results presented herein pave the way for future studies aimed at understanding the roles of these proteases in IBD pathophysiology. This study also revealed strong differences between CD patients and UC in terms of profiles of active proteases that could dictate distinct and specific therapies for these 2 sub-categories of IBD patients in the forthcoming targeted therapy strategies. The approach proposed here can be applied to other diseases and other tissues, in order to identify active secreted proteases that may play roles in other inflammatory diseases, so as to serve as potential new therapeutic targets.

## Methods

### Patients and colonic samples

This work and tissue collection received ethical approval. All methods were performed in accordance with guidelines and regulations from the French Ethics Committee (Comité d’Ethique sur les Recherches Non Interventionnelles) (Identifier: NCT01990716). Colonic tissue samples were obtained from well-characterized CD and UC patients undergoing colonoscopy or colonic resection procedures at the Toulouse Hospital Centre (France). Colonic tissue samples from individuals undergoing colon cancer screening who were otherwise healthy were used as controls. Written and verbal informed consent was obtained before enrolment in the study. Fresh colonic tissue samples were rinsed in isotonic sterile Hanks’ balanced salt solution pH 7.4 (HBSS) and were then immediately incubated in 2 ml of HBSS (containing Ca^2+^ and Mg^2+^) at 37 °C for 60 min. Freshly isolated colonic tissue specimens were quick-frozen in RP1 buffer (Macherey-Nagel, GmbH) and stored at −80 °C until use for RNA extraction.

### Measurement of protein concentration

The concentration of protein in colonic tissue supernatants was determined by using the Pierce Protein BCA Assay Kit, according to instructions (Thermo Scientific).

### Measurement of proteolytic activity

The proteolytic activity was measured in colonic tissue supernatant samples with 0.1 mM N-p-Tosyl-GPR-amino-4-methylcoumarin hydrochloride as substrate in 50 mM Tris, 10 mM CaCl_2_, pH = 8 (Sigma-Aldrich)^29^. Substrate cleavage was calculated by the change in fluorescence (excitation: 355 nm, emission: 460 nm), measured over 30 min at 37 °C on a Varioskan Flash microplate reader (Thermo Fisher Scientific). Sample values were interpolated into a linear regression generated with a standard curve of TPCK-treated trypsin from bovine pancreas (8–500 mU/mL; Sigma-Aldrich). Data were expressed as mU of trypsin-like activity per mg of protein.

### Activity-Based Probe reaction

The Biotin-PK-DPP serine protease activity-based probe was obtained from the laboratory of Dr. Nigel W. Bunnett (Columbia University, USA), with the participation of Dr. Laura Edgington-Mitchell (Monash University, Australia) and synthetized as previously described^30^. This probe presents a relative selectivity towards Enzyme Class 3.4.21.4 and EC 3.4.21.5^16,30^. Colonic supernatants (40 µg of protein) were diluted in 100 mM Tris-HCl, 1 mM CaCl_2_, pH = 8 to a final volume of 900 µL and then split into duplicates. Each duplicate (450 µL) was then pre-treated or not with a final concentration of 4 mM AEBSF (SIGMA) during 15 min at 37 °C under stirring (1000 rpm). The pre-incubation with this irreversible broad-spectrum serine protease inhibitor allows the identification of active proteases, since enzyme inhibition abrogates their interaction with the ABP, impacting the intensity signal of bands in proteomic profiles and of peptides retrieved by mass spectrometry. Then, the ABP biotin-PK-DPP was added to each reaction to a final concentration of 1 μM, and each replicate sample, containing 20 ug of protein, was incubated for 60 min at 37 °C under stirring (1000 rpm).

### Functional proteomic profiling

The reaction product was then precipitated in 15% trichloroacetic acid at 4 °C during 90 min. The pellet was washed twice in cold acetone (−20 °C) and solubilized in 20 μL of protein solving buffer with tris-(2-carboxyethyl)-phosphine hydrochloride (PSB-TCEP; Macherey-Nagel, GmbH). Samples were then heated at 95 °C for 5 min, clarified by centrifugation at 12000 × *g* for 5 min and the solubilized sample was loaded into 4–20% Mini-Protean TGX precast gels (Bio-Rad, GmbH). After electrophoresis, the proteins were blotted onto nitrocellulose membranes by using the Trans-Blot Transfer Turbo System (Bio-Rad). Membranes were incubated with streptavidin-HRP (Life Technologies), and bands were visualized with ECL Prime Western Blot Detection Reagent (GE Healthcare Life Sciences) and quantified by chemiluminescence yield (Chemidoc XRS; Bio-Rad). The molecular weight and intensity of each band was determined with the Image Lab Software v5 (Bio-Rad). The bands corresponding to active proteases were identified by their sensitivity to AEBSF. Additionally, the activity index of each protease-corresponding band was estimated by the calculation of a ratio between the volumetric densitometry of the fluorescent signal generated by untreated *vs* AEBSF-pretreated duplicates (−/+AEBSF). An activity index of 0 was given to cluster bands that were not detected in specific samples.

### Mass spectrometry analysis

For mass spectrometry analysis, colonic supernatants from 3 representative patients per group were pooled and submitted to an ABP reaction in a final volume of 4.0 mL, as described above. In the following, 3.8 mL of the reaction product were incubated with 50 μL of pre-washed Dynabeads MyOne Streptavidin C1 (Invitrogen, USA) for 60 min at room temperature under stirring (1000 rpm). The beads were washed 5 times with 1 mL of phosphate buffered saline pH = 7.2. As a control procedure for the ABP reaction and following steps, 200 μL of ABP-labelled secretome fluids (before incubation with beads), bead supernatant and buffer from the first wash were recovered, precipitated and analysed by proteomic profiling. The pellets containing the ABP-protease complexes adsorbed to the magnetic beads were washed twice with 50 mM ammonium bicarbonate buffer (Sigma-Aldrich, USA), and then suspended in 6 M urea and 25 mM DTT (Sigma-Aldrich). After 60 min under stirring (850 rpm) at room temperature, the samples were alkylated by the incubation in 90 mM iodoacetamide (Sigma-Aldrich) during 30 min in the dark. Bead-bound samples were then washed twice as described above and submitted to overnight proteolysis at 37 °C in ammonium bicarbonate buffer (50 mM, pH = 8.5) containing 1 μg of trypsin (Promega, USA) per sample. The supernatants were collected, dried under vacuum and solubilized in 2% acetonitrile and 0.05% trifluoroacetic acid (Sigma-Aldrich), for further analysis.

The resulting peptides were analysed with a NanoLC (Ultimate 3000 RSLCnano system Thermo Scientific) coupled to a LTQ Orbitrap Velos mass spectrometer (Thermo Fisher Scientific, Bremen, Germany). Peptides extracts (5 μL) were loaded on a C18 precolumn (300 μm inner diameter x 5 mm; Thermo Scientific) in a solvent made of 2% acetonitrile and 0.05% trifluoroacetic acid, at a flow rate of 20 μl/min. After 5 min of desalting, the precolumn was switched online with the analytical C-18 column (75 μm inner diameter x 50 cm; Reprosil) equilibrated in 95% of solvent A (0.2% formic acid) and 5% of solvent B (80% acetonitrile and 0.2% formic acid). The peptides were eluted using a 5–50% gradient of solvent B over 105 min at a flow rate of 300 nL/min. The LTQ Orbitrap Velos was operated in a data-dependent acquisition mode with Xcalibur software. MS survey scans were acquired in the Orbitrap on the 350–1800 *m/z* range, with the resolution set to 60,000. The 20 most intense ions per survey scan were selected for fragmentation by collision-induced fragmentation and MS/MS spectra were acquired in the linear ion trap. A 60 s dynamic exclusion was used to prevent repetitive selection of the same peptide. Triplicate LC-MS measurements were performed for each sample.

### Protein identification and quantification

Raw MS files were processed with MaxQuant software (version 1.5.2.8) for database search with the Andromeda search engine and for quantitative analysis. Data were searched against *human* entries in the Swissprot protein database (release UniProtKB/Swiss-Prot 2015–12; 20200 entries). Carbamidomethylation of cysteine was set as a fixed modification, whereas oxidation of methionine, protein N-terminal acetylation were set as variable modifications. Specificity of trypsin digestion was set for cleavage after K or R, and two missed trypsin cleavage sites were allowed. The precursor mass tolerance was set to 20 ppm for the first search, 5 ppm for the main Andromeda database search and minimum peptide length was set to 7 amino acids. Andromeda results were validated by the target-decoy approach using a reverse database at both a peptide and a protein false-discovery rates of 1%. For label-free relative quantification of the samples, the match between runs option of MaxQuant was enabled with a time window of 0.7 min, to allow cross-assignment of MS features detected in the different runs.

To perform relative quantification between proteins identified, we used the “Intensity” metric from the MaxQuant “protein group.txt” output (sum of peptide intensity values for each protein). Quantitative data were first normalized and missing protein intensity values were replaced by a constant noise value that was determined independently for each sample as the lowest value of the total protein population. Enrichment ratios between AEBSF not treated and AEBSF treated samples were calculated from the mean protein intensities derived from three technical replicate experiments. A potential active protease was selected based on an enrichment ratio >2 (Intensity AEBSF not treated vs. treated) and a Student’s t-test P-value < 0.05 over the triplicates.

### RT-PCR

Total RNA was extracted from human colonic tissue samples with the Nucleospin RNA/Protein Kit (Macherey-Nagel, GmbH). DNAse-treated RNA was reverse transcribed using the Maxima First Strand cDNA Synthesis Kit (Thermo Scientific). Resulting cDNA samples were amplified by conventional PCR with Taq DNA polymerase (Invitrogen, USA) and sequence-specific primer pairs (Supplementary Table 1). As a control procedure, RT reactions were performed without the addition of enzyme to the mix.

### Statistical analysis

For the enzyme kinetics and proteomic profiles assays, each dot represents the data from an individual patient. All data were used to calculate the values expressed as mean ± SEM. Statistical analysis was performed using One-Way Analysis of Variance (ANOVA) or Kruskal-Wallis, followed by multi comparison tests, as indicated in figure legends. Outliers were identified by the method of ROUT with Q settled at 1%. GraphPad Prism v.6 software was used for analysis. Statistical significance was accepted at p < 0.05.

### Data Availability

The mass spectrometry proteomics data have been deposited to the ProteomeXchange Consortium via the PRIDE^31^ partner repository with the dataset identifier PXD009450.

## Electronic supplementary material

supplementary figures
