# Supplementary material for: Functional Proteomic Profiling of Secreted Serine Proteases in Health and Inflammatory Bowel Disease
Source: Sci Rep. 2018 May 18;8:7834. doi: 10.1038/s41598-018-26282-y (PMC5959920; doi:10.1038/s41598-018-26282-y)
Supplement: Supplementary file 1 — supplementary figures [file 41598_2018_26282_MOESM1_ESM.pdf]

Functional Proteomic Profiling of Secreted Serine Proteases in Health and Inflammatory Bowel Disease

Alexandre Denadai-Souza; Chrystelle Bonnart; Núria Solà Tapias; Marlène Marcellin; Brendan Gilmore; Laurent Alric; Delphine Bonnet; Odile Burlet-Schiltz; Morley D. Hollenberg, Nathalie Vergnolle; Céline Deraison

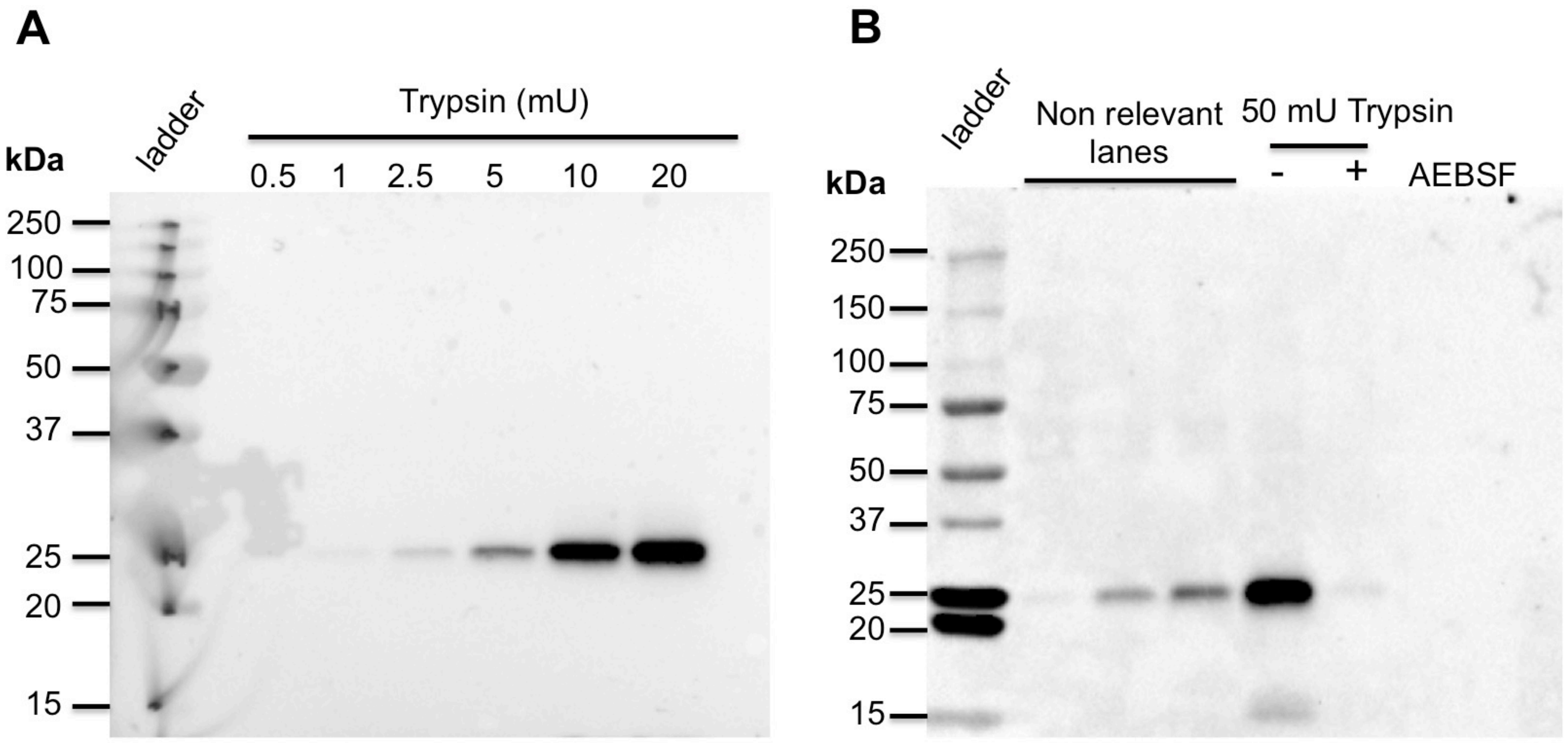

Supplemental Figure 1. Full-length gels of Figure 1. Pictures correspond to merged pictures between the luminescent signal and colorimetric signal, in order to have the ECL signals and the prestained molecular weight ladder present on the same picture. In the cropped figure 1, only ECL signal picture is depicted, this might explain difference in background, notably the presence of smudge in panel A which is not present in the cropped corresponding panel A in Figure 1.

Functional Proteomic Profiling of Secreted Serine Proteases in Health and Inflammatory Bowel Disease

Alexandre Denadai-Souza*[1]; Chrystelle Bonnart*[1]; Núria Solà Tapias[1]; Marlène Marcellin[2]; Brendan Gilmore[3]; Laurent Alric[4]; Delphine Bonnet[1]; Odile Burlet-Schiltz[2]; Morley D. Hollenberg[5], Nathalie Vergnolle[#$1,5]; Céline Deraison[#1]

Supplementary Table 1.

Transcript	Sequences (5'-3')	Amplicons (bp)	Splice variant	Accession number
CTSG	TGAGAGTGCAGAGGGATAGG	154		NM_001911
	AAGCCATTGTCACCCCAG			
CELA3A	CTTTGGCTGCAACTTCATCTG	141		NM_005747
	TCTTTATTCAGGATGTGGGATCG			
F2	GAGGACGCCTCGAGATAAGC	297	1	NM_000506
	GTGACTTGATCCTGGCCACA	297	2	NM_001311257
KLKB1	TCTTGCGTTCTCAGATGTGG	256	1	NM_000892
	ATGGCAGGGTTCAGGTAAAG	256	2	NM_001318394
		287	3	NM_001318396
PLG	AAGAGTCCAATCCACCGAAC	290		NM_000301
	CATGCTAAATCCCTACCCACG			
TPSAB1/TPSB2	CTGGCATCTACACCCGTG	143		NM_003294
	TGGGTAGGAAGCAGTGGT			NM_024164
RNPEP	AGAACCCTTGTCTGACCTTTG	263	1	NM_020216
	CTCTCCAGTGATGTCCATGTG	263	2	NM_001319182
		263	3	NM_001319183
		263	4	NM_001319184

Supplementary Table 1. Sequences of oligonucleotides used for RT-PCR experiments. The human gene symbols, oligonucleotide sequences, amplicon size in base pairs (bp), individual or multiple splice variants targeted by each primer pair and respective NCBI accession numbers are indicated.
